# Characterization of the Materials Synthesized by High Pressure-High Temperature Treatment of a Polymer Derived t-BC_2_N Ceramic

**DOI:** 10.3390/ma4122061

**Published:** 2011-11-29

**Authors:** Wallace R. Matizamhuka, Iakovos Sigalas, Mathias Herrmann, Leonid Dubronvinsky, Natalia Dubrovinskaia, Nobuyoshi Miyajima, Gabriela Mera, Ralf Riedel

**Affiliations:** 1 E6 Advanced Materials, Element Six (Production) (Pty) Ltd, 1 Debid Road Nuffield, Springs 1559, South Africa; 2 School of Chemical & Metallurgical Engineering, University of the Witwatersrand, P.Bag 3, Johannesburg Wits 2050, South Africa; E-Mail: iakovos.sigalas@wits.ac.za; 3 DST/NRF Centre of Excellence in Strong Materials, Physics Building, University of the Witwatersrand, Johannesburg 2000, South Africa; 4 Fraunhofer Institute for Ceramic Technologies & Systems, Winterbergstr. 28, Dresden 01277, Germany; E-Mail: mathias.herrmann@ikts.fhg.de; 5 Bayerisches Geoinstitut, Universität Bayreuth, Universitätstr. 30, Bayreuth 95447, Germany; E-Mails: leonid.dubrovinsky@uni-bayreuth.de (L.D.); nobuyoshi.miyajima@uni-bayreuth.de (N.M.); 6 Materialphysik und Technologie, Lehrstuhl für Kristallographie, Universitüt Bayreuth, Universitätstr. 30, Bayreuth 95447, Germany; E-Mail: natalia.dubrovinskaia@geow.uni-heidelberg.de; 7 FG Disperse Feststoffe, Institut für Materialwissenschaft, Technische Universität Darmstadt, Petersenstr. 32, Darmstadt 64287, Germany; E-Mails: mera@materials.tu-darmstadt.de (G.M.); riedel@materials.tu-darmstadt.de (R.R.)

**Keywords:** boron carbonitride, polymer-derived ceramics, sintering, spectroscopy, X-ray diffraction

## Abstract

Bulk B-C-N materials were synthesized under static high thermobaric conditions (20 GPa and 2,000 °C) in a multianvil apparatus from a polymer derived t-BC_1.97_N ceramic. The bulk samples were characterised using X-ray synchrotron radiation and analytical transmission electron microscopy in combination with electron energy loss spectroscopy. Polycrystalline B-C-N materials with a cubic type structure were formed under the applied reaction conditions, but the formation of a ternary cubic diamond-like c-BC_2_N compound, could not be unambiguously confirmed.

## 1. Introduction

In recent years, several studies have been dedicated to the exploration of the B-C-N phase system [1,2,3,4,5,6,7,8,9,10]. The interest has been due to the expectation of its unique properties such as extreme hardness, chemical inertness and semi-conducting properties for diamond-like high pressure phases [1,2,3,4,5,6,7]. In particular, the metastable c-BC_2_N has been obtained under high static pressure (≥20 GPa) and high temperature (HP/HT) with a Vicker’s microhardness of 76 GPa [7]. Such materials would be complimentary to diamond and c-BN for high speed ferrous alloy cutting and polishing applications.

Several methods have been employed in fabricating graphite like BN-C compounds such as chemical vapour deposition (CVD), where typically BCl_3_/CH_3_CN gaseous mixtures have been used [8,9,10,11,12,13,14,15], mixing and ball milling of graphite/h-BN powders [4,5,16,17,18,19], solid phase nitriding [20] and solid state pyrolysis [21,22,23,24,25]. However, the progress of most of the methods used so far has been hindered by either inability to produce complete BC_x_N solid solutions or inability to produce them in high yields. This is partially because most of the precursors used so far provided inhomogeneous product mixtures.

One of the most important factors in the successful HP/HT synthesis of a cubic BC_x_N lies in the quality of the starting materials used [1,2,3,4,5,6,7]. In the pioneering work of Riedel *et al.* [21,23,24] an extensive study on polymer derived BN-C materials showed the potential of obtaining boron carbonitrides in high ceramic yields from boron-based polymers by thermal decomposition under a controlled atmosphere.

In the present study we carried out static HP/HT synthesis using a specific polymer derived turbostratic-BC_2_N (t-BC_2_N) ceramic to assess its potential as a source for the high pressure cubic BN-C materials. Turbostratic-BC_2_N may be considered as an intermediate between the crystalline h-BC_2_N and the amorphous state and possesses random translation of layers along the c-axis. Typical X-ray diffraction patterns of the turbostratic type structure reveal the presence of (*hk0*) and (*00l*) reflections. The general crystalline type (*hkl*) reflections are missing [26]. Due to lack of order in the turbostratic phase, the transformation to the cubic phase would be expected to take place under milder conditions in comparison to the h-BC_2_N to c-BC_2_N conversion. This expectation is due to the fact that the h-BC_2_N structure has to pass through some form of disorder during transformation [27].

## 2. Experimental Section

The BN-C starting materials used in the present study were synthesized by the solid state pyrolysis of a boron containing polymer derived from piperazine borane, C_4_H_10_N_2_·BH_3_, as reported in literature [23,24,25]. Piperazine borane was obtained by the reaction of piperazine (99%) with borane dimethyl sulphide complex in a molar ratio of 1:1. The reaction product, C_4_H_10_N_2_·BH_3_, is formed in 87% w/w yield as a fine white powder. The polymerisation of the piperazine borane occurs at 400 °C (10 h in Ar atmosphere) and the product is isolated as a yellow coloured powder [23]. The pyrolysis was done under N_2_ at temperatures of 1,450 °C, 1,650 °C and 1,850 °C with a residence time of 2 h. The composition of the pyrolysed (1,050 °C) and heat treated (1,850 °C) products are shown in Table 1.

**Table 1:** Chemical composition of the as-pyrolysed (Pyro-BN-C at 1,050 °C, 2 h, N_2_) and the heat-treated (Heat-treat-BN-C at 1,850 °C, 2 h, N_2_) ceramic obtained by CNO elemental analysis.

Sample	C (w/w %)	N (w/w %)	C/N ratio	O (w/w %)
Pyro-BN-C	40.0 ± 0.4	22.9 ± 1.1	1.75	4.2 ± 0.3
Heat-treat-BN-C	46.4 ± 0.6	23.5 ± 2.0	1.97	0.4 ± 0.2

X-ray diffraction was carried out using CuKα radiation with a step scan of 0.02° in the 2θ regime of 10–80° with a Philips PW1780 diffractometer. The bulk elemental analysis was done using the combustion (Leco C-200) and inert gas fusion methods (Leco TC-436). The surface morphology of the powders was analyzed using a LEO-1530 SEM (Scanning Electron Microscope).

Static HP/HT syntheses were conducted in 1,200 t *Sumitomo* and large volume 5,000 t *Zwick-Voggenreiter* “6–8” type multi-anvil presses at the Bayerisches Geoinstitut. The samples were contained in either h-BN or Ta capsules to avoid chemical reactions at elevated temperatures. The sample assembly consisted of a MgO (+*ca.* 5 w/w % Cr_2_O_3_) octahedron and a LaCrO_3_ heater surrounding the sample capsule with Mo discs at both ends of the heater for electrical conductivity. The assembly was placed in an octahedron cavity created between eight WC cubes each with a truncation of 11 mm or 5 mm for the 5,000 t and 1,200 t assemblies respectively. Each experiment consisted of compressing the assembly to the desired pressure, followed by a subsequent temperature increase at a rate of 100 °C/min and an isothermal holding time of 30 s to 5 min at the target temperature. The samples were subsequently quenched by switching off the furnace power. The octahedra were carefully opened and samples mechanically cleaned in acetone using an ultrasonic bath for about 30 min. The samples designated **S** and **Z** were synthesised using the 1,200 t ***S****umitomo* press and the 5,000 t ***Z****wick-Voggenreiter* presses respectively and the numerals refer to the experiment numbers.

The materials were initially studied with a Rigaku FR-D high-brilliance X-ray diffractometer (MoKα radiation, *λ* = 0.7108 Å). Rotational diffraction patterns were collected for 1 h at room temperature. The 2-D diffraction patterns obtained were corrected for spatial distortions and integrated using the FIT2D software [28]. Additionally, synchrotron radiation facilities at the ESRF, Grenoble, France (*λ* = 0.7693 Å) and APS Argonne National Laboratory Chicago, USA (*λ* = 0.3344 Å) were used for further analysis. The lattice parameters were determined using a GSAS Software package [29].

In order to ascertain the morphology, composition and the bonding nature in the material, the HRTEM microscopy combined with Electron Energy Loss Spectroscopy (EELS) technique was performed. The samples were crushed between WC anvils, dispersed in ethanol and loaded onto copper grids coated with a holey carbon film. A Philips CM-20 FEG (Field Emission Gun) TEM operated at 200 kV was used in the present study. The microstructures were studied by both BF (bright field) and DF (dark field) imaging and HRTEM (High Resolution Transmission Electron Microscopy). Furthermore, when possible and appropriate SAED (Selected Area Electron Diffraction) was used to obtain the interplanar spacing of the three strongest diffraction rings 111, 220 and 311 which were subsequently used for lattice constant (*a*) determination. In an attempt to ascertain the nature of bonding in the cubic phase, we collected EELS data of the B-K, C-K and N-K edges using a GATAN-PEELS 666 (parallel electron energy loss spectrometer) on a selected area. The relative compositions were obtained from:

(1) $$\frac{C_{A}}{C_{B}}=\frac{\sigma_{K}^{B}(\beta ,∆E)*I_{K}^{A}(\beta ,∆E)}{\sigma_{K}^{A}(\beta ,∆E)*I_{K}^{B}(\beta ,∆E)}$$

where *C_A_* and *C_B_* are atomic concentrations of elements A and B respectively and $\sigma_{K}^{A}$ $\sigma_{K}^{B}$ are the partial ionisation cross sections for *K_th_* ionisation edges evaluated over the spectrometer acceptance angle β with energy window *∆E*. (with collection angles (2β) of 2.9 mrad or 4.3 mrad and convergence angle (2α) of 8 mrad). $I_{K}^{A}$ and $I_{K}^{B}$ are the intensities of the *K* ionisation edges.

## 3. Results

### 3.1. Precursor Material

The formation of BC_2_N from the piperazine-borane, C_4_H_10_N_2_·BH_3_ takes place through the release of H_2_, CH_4_ and NH_3_ according to the quantitative reaction below:

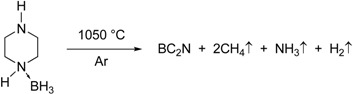


**Figure 1:**
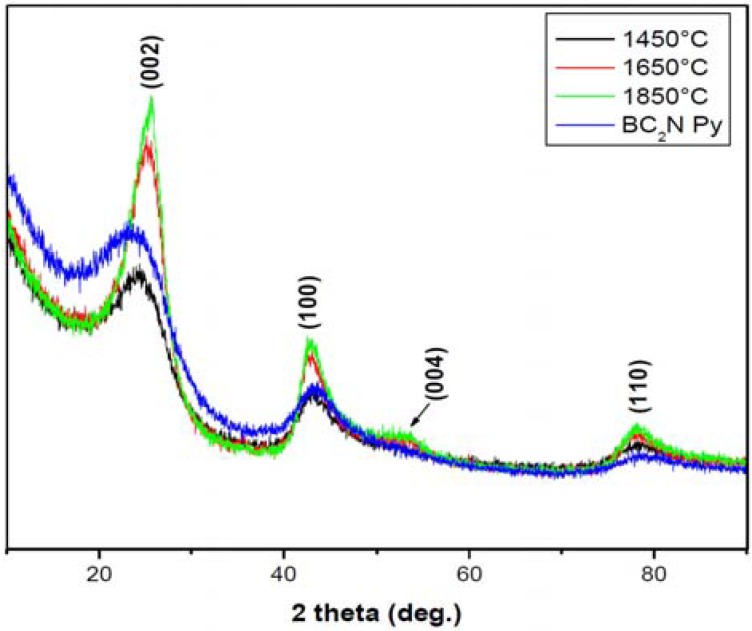
X-ray diffraction patterns of the BN-C materials heat treated at different temperatures, BN-C Py refers to the material pyrolysed at 1,050 °C (Y-scale is the intensity in a.u.).

Nominal compositions of BC_1.75_N and BC_1.97_N were obtained for the pyrolysed (1,050 °C) and heat treated (1,850 °C) powders (Table 1). There was minimal oxygen contamination after heat treatment up to 1,850 °C. The as-pyrolysed BN-C materials are X-ray amorphous showing broad diffuse turbostratic diffraction lines. There is a noticeable decrease in the peak widths and increased intensities with increasing temperature, the ceramic shows low crystallinity even at higher temperatures as shown in Figure 1.

**Figure 2:**
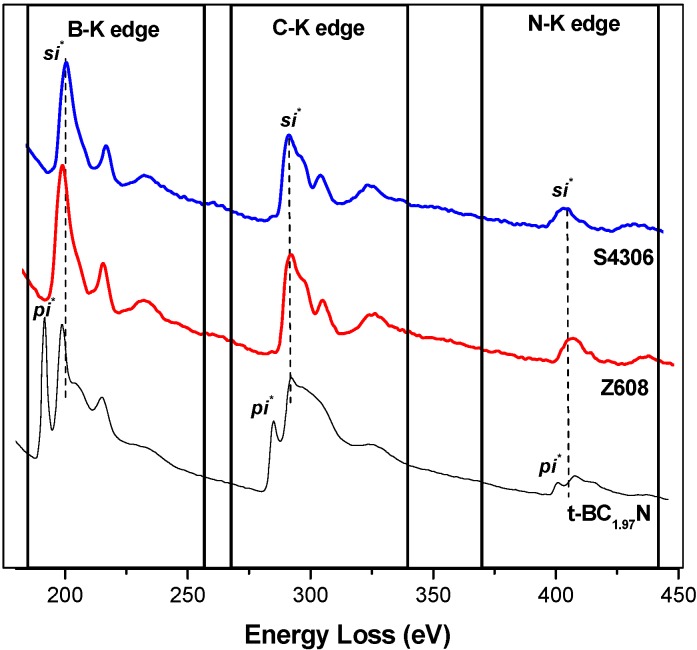
Electron Energy-Loss Spectrum (EELS) of the starting t-BN-C material (heat treated at 1,850 °C) in comparison to the material treated at high pressure and temperatures (samples S4306 (20 GPa/2,000 °C/30 s) and Z608 (20 GPa/2,000 °C/60 s)).

**Figure 3:**
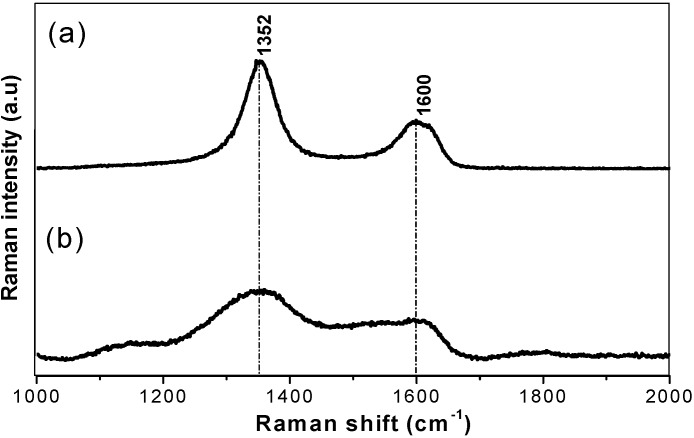
First order Raman spectra of heat treated at 1,850 °C (**a**) in comparison to the as-pyrolysed (1,050 °C) BC_2_N ceramic (**b**).

The EELS profile (Figure 2) of the heat treated (1,850 °C) starting BN-C ceramic shows the B-K, C-K and N-K edges appearing concurrently throughout the whole spectrum. Pre-edge features at 191.4 eV and 197.7 eV at the B-K edge, 285 eV and 292.5 eV at the C-K edge and 400 eV and 407.7 eV at the N-K edge were identified as transitions due to π* (pi*) and σ* (sigma*) molecular orbitals confirming the sp^2^ type bonding in the starting materials. The peaks occurring at higher energy loss values in each K-edge range are featureless. It should be noted that at the carbon ionisation edge the EELS spectra of both HP synthesised samples closely resemble those of diamond, as well as those of cBN at the boron and nitrogen ionisation edges. As summarised in Table 2, the results were compared with those of graphite, h-BN and g-BC_2_N obtained in previous studies. The EELS profile is consistent with that of g-BC_2_N and different from those of well crystallised graphite or h-BN. The Raman spectra of the BN-C ceramics are characterised by broad D (1,352 cm^−1^) and G (1,600 cm^−1^) bands (Figure 3) which confirms the existence of C-C type bonding.

**Table 2:** A summary of the energy loss values of the present t-BN-C materials in comparison to those of pure h-BN, graphite, graphitic and cubic B-C-N material. The energy positions of the π* and σ* peak maximums in C-K spectra were assumed to be at 285 eV and 291.7 eV, respectively.

Sample	B-K(eV)		C-K (eV)		N-K (eV)		Reference
	π*	σ*	π*	σ*	Π*	σ*	
g-BC_2_N	191.6	197.7	285	291.7	400.7	406.3	[30]
t-BN-C	191.3	198.9	284.9	292.5	401.2	407.4	Present work
h-BN	191.9	198.1	-	-	401.6	408.1	[30]
graphite	-	-	285	291.7	-	-	[30]
Z608	-	196.9	-	291.7	-	407.5	Present work
S4306	-	196.1	-	291.7	-	406.3	Present work
diamond	-	-	-	291.7	-	-	[30]
c-BN	-	197.7	-	-	-	406.3	[30]

### 3.2. High Pressure Synthesised Materials

**Figure 4:**
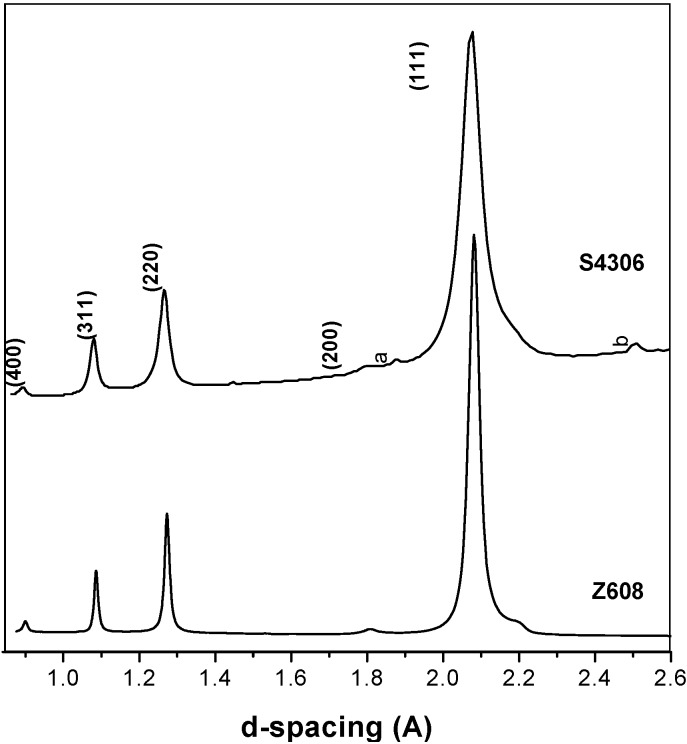
XRD patterns of samples S4306 and Z608 taken with monochromatic synchrotron radiation. Two minor reflexions labelled *a* and *b* and the shoulders of the main peak in the Z608 sample do not correspond to the cubic reflections.

The results in Table 3 show that cubic type phase(s) were obtained at 20 GPa and 2,000 °C signifying transformation occurred at the synthesis conditions specified. At a reaction temperature of 1,920 °C some starting t-BN-C material still existed in the final product and no conversion was observed at lower temperatures (e.g., at 1,500 °C or at room temperature). The XRD data of the transformed material confirmed the presence of a phase with a cubic diamond- or sphalerite-type structure (Figure 4). Furthermore, two peaks were observed at high *d*-values (*a* = 2.19(7) Å and *b* = 1.88(5) Å, Figure 4) for sample *S4306* which did not match any of the suspected compounds (*i.e*., Ta, TaB_x_, TaC_x_ generated through possible reactions with the capsule material used), but were rather close to reflections of wurtzite-structured BN. For comparison, the parameters and results obtained in previous studies are listed in Table 3.

**Table 3:** Summary of synthesis HP/HT conditions, final products, and refined lattice parameters (*GSAS Software Package*) [29] of the present B-N-C materials in comparison to previously obtained c-BC_2_N phases.

Sample/Starting material	P [GPa]	T [°C]	Holding time [s]	Products	Lattice parameter [Å]	Method	Reference
Z608	20	2,000	60	Diamond-like phases	3.6006(3)	MA	Present work
S4306	20	2,000	30	Diamond-like phases	3.596(6)	MA	Present work
S4295	20	1,920	300	t-BN-C + Diamond-like phases	-	MA	Present work
S4294	20	1,500	120	t-BCN	-	MA	Present work
S4311	20	25	7,200	t-BCN	-	MA	Present work
Milled BN/C_2_	20	1,927	300	c-BC_2_N	3.595(7)	MA	[5]
g-BC_2_N	25	1,820	1,800	c-B_0.4_C_1.1_N_0.5_	3.642(2)	MA	[7]
g-BC_2_N	50	3,227	10^−6^	c-BC_2.5_N	3.605	SC	[27]
Milled BN/C_2_	30	1,727	300–600	c-(BN)_0.67_C_0.33_	3.613(3)	LHDAC	[4]
g-BC_2_N	7.7	2,300	900	c-BN, Diamond c-B/C/N mixture	-	BP	[3]

MA: multianvil press; SC: Shock Compression; BP: Belt Press; LHDAC: Laser Heated Diamond Anvil Cell. Microhardess quoted in reference [7] is 76 GPa, lattice parameters obtained from Synchrotron XRD results.

As is stated above, the major diffraction peaks of Z608 are consistent with a sphalerite-type structure (Figure 4). A lattice parameter of *a* = 3.6006(3) Å was obtained using full-profile refinement. The lattice parameter and the presence of (200) reflection (~1.800 Å) suggest that the material contains mainly c-BN. Indeed, for pure c-BN *a* = 3.616 Å (*JCPDS no.* 35–1365), and for diamond *a =* 3.567 Å (*JCPDS no.* 6–0675). The unit cell parameter is slightly smaller than expected for c-BN and this fact may be related to the following; residual stresses in the sample, the crystallite size [31], possible dissolution of a certain amount of carbon (about 30%) in the c-BN structure, or to the presence in the sample of a small amount of (nano- or boron doped) diamond [32,33,34,35]. Diamond reflections, although not resolved from those of c-BN, are effectively shifting the centre of the observed reflection to higher 2θ angles.

**Figure 5:**
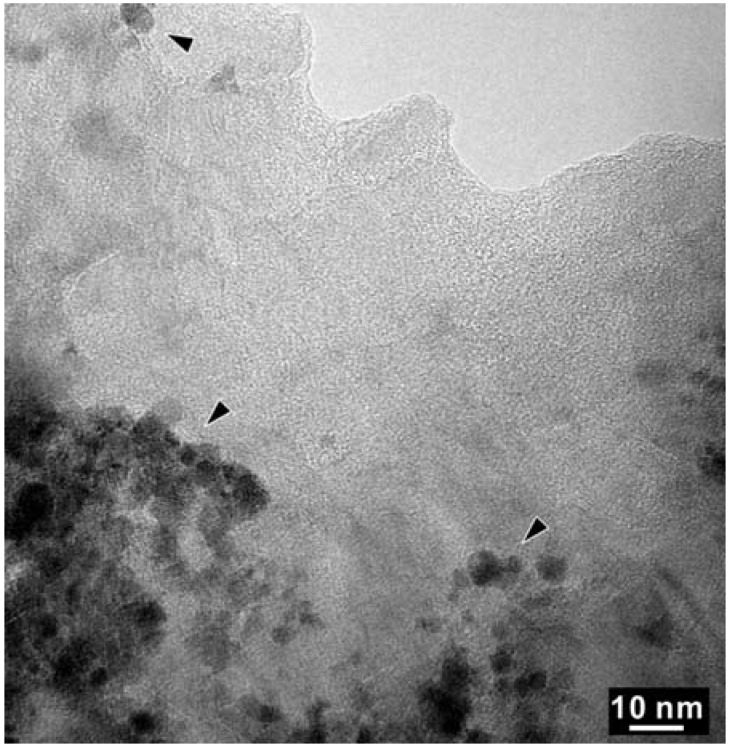
High Resolution TEM (HR-TEM) of a selected area of the BN-C domain of sample Z608. The arrowed regions with darker contrast were found to contain only C-K edges in EELS.

**Figure 6:**
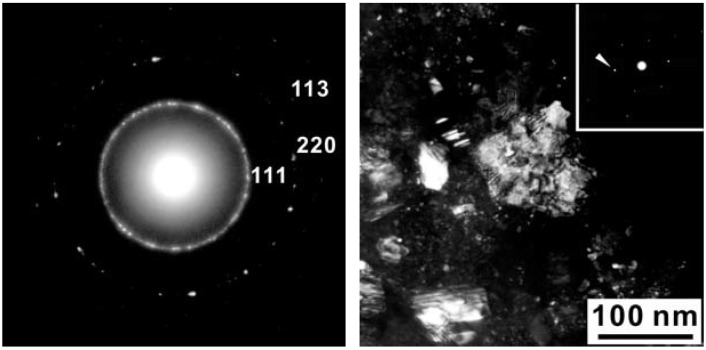
(**a**) Selected Area Electron Diffraction (SAED) pattern of sample Z608 in the area of the B-C-N phase; (**b**) Dark field image with g = 1 0 $\bar{1}$ 1 (cubic setting) or 1 1 1 (cubic setting) which are indicated by the arrow in the SAED (inset). Selected area electron diffraction of sample Z608 showing a nano-sized crystallite.

As shown in Figure 5, High Resolution TEM and EELS revealed the decomposition of the t-BC_2_N phase in the Z608 sample resulting in nano-diamond and nano B-N-C material. While the presence of nano-diamond is confirmed by selected area electron diffraction (SAED) by observation of (111), (220), and (311) reflections, the portion of the sample containing all three elements (e.g., B, N and C) can not be unambiguously attributed to the sphalerite-type structure (Figure 6).

**Figure 7:**
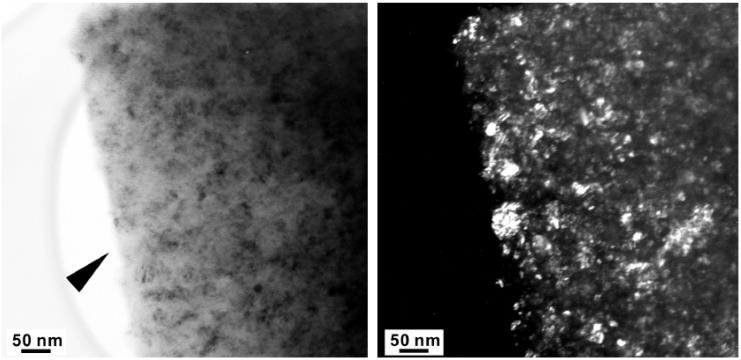
Bright field (BF) (left) and dark field (DF) (right) images of sample S4306 showing nano-sized polycrystalline material.

**Figure 8:**
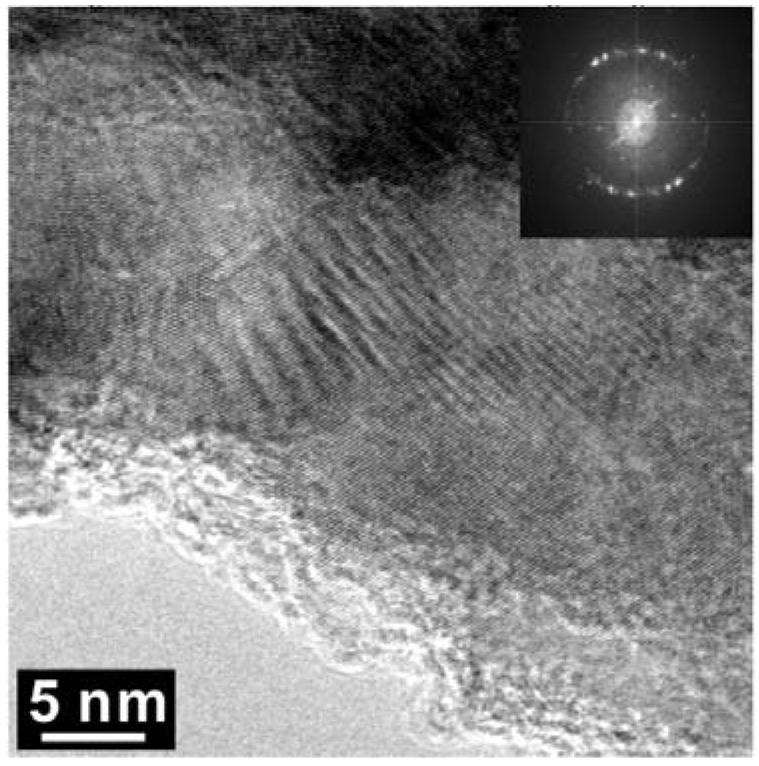
High Resolution-TEM micrograph of the arrowed region in Figure 5. The insert is an FFT (fast Fourier transform) pattern.

The major diffraction peaks of S4306 are consistent with a sphalerite-type structure. The only difference is the presence of additional peaks observed in S4306. A unit cell parameter of a cubic phase is *a =* 3.596(7) Å, coinciding within uncertainties with the value obtained for the Z608 sample. Bright field (BF) and dark field (DF) imaging of the S4306 sample show relatively fine aggregated polycrystalline materials with approximate particle sizes of 5–10 nm (Figure 7). An FFT (Fast Fourier Transform) pattern of the arrowed point in Figure 8 shows discrete spots, further confirming the existence of extremely fine crystallites (Figure 8). Single crystal diffraction on such extremely small crystallites is not possible. The EELS spectrum from a selected area confirms the presence of B, C, and N atoms with sp^3^ hybridised bonds, but due to size of the small crystallites it can not distinguished, if these atoms belong to a ternary compound or to a mixture of nano-diamond and nano-cBN (2,5).

## 4. Discussion

Characterisation of the precursor material shows a homogeneous turbostratic BN-C material with a nominal composition of BC_1.97_N. The Raman spectrum of the precursor material does not resemble that of pure graphite (1,581 cm^−1^) or graphite-like BN (1,366–1,370 cm^−1^) ruling out phase separation. Furthermore, EELS of the starting material shows the existence of sp^2^ hybridisation with both π and σ bonding. A complete transformation of the turbostratic precursor into phases with sp^3^-bonded B, N, or C atoms occurred at a temperature of 2,000 °C and pressure of 20 GPa as shown by X-ray diffraction and EELS results. The identified phases are nano-diamond and nano-C-B-N material, while the existence of ternary compound(s) with the cubic structure can not be unambiguously confirmed. The experiment at a temperature of 1,920 °C (*S4295*) with a longer isothermal holding time at 20 GPa resulted in incomplete transformation, which is a clear indication of the importance of a sufficiently high temperature. Lowering the treatment temperature down to 1,500 °C resulted in no transformation (*S4294*). The room temperature experiment (*S4311*) at 20 GPa with 7,200 s of the isobaric holding time clearly rules out the possibility of the HP transformation without temperature assistance. It is therefore apparent that the direct synthesis of a bulk nano cBN/nano-diamond composite ceramic from a homogeneous polymer derived BN-C precursor still requires similar high thermobaric conditions to those used in previous studies, despite the good homogeneity and the low crystallinity of the precursor materials. EELS data shows that the bonding nature changes from an sp^2^ to sp^3^ type hybridisation only during high pressure high temperature treatment.

## 5. Conclusions

It has been shown that a homogeneous turbostartic-BC_2_N phase can be produced by the polymer-to-ceramic transformation route. The turbostratic phase is a suitable precursor for the synthesis of nanostructured bulk B-C-N materials. X-ray diffraction and TEM/EELS results reveal a nano-cBN and nano-diamond in products recovered after high-pressure high-temperature treatment. It is apparent that the direct synthesis of a hard nanocrystalline B-C-N ceramic from polymer derived BC_2_N requires high thermobaric conditions (T > 1,920 °C at 20 GPa) despite the good homogeneity and the low crystallinity of the precursor used in this investigation.
